# Barriers and Enablers for Healthy Food Systems and Environments: The Role of Local Governments

**DOI:** 10.1007/s13668-022-00393-5

**Published:** 2022-02-12

**Authors:** Nicholas Rose, Belinda Reeve, Karen Charlton

**Affiliations:** 1 Faculty of Higher Education, William Angliss Institute, Melbourne, Victoria Australia; 2 The University of Sydney Law School, Sydney, NSW Australia; 3 School of Medical, Indigenous and Health Sciences, Faculty of Science, Medicine and Health, The University of Wollongong, Wollongong, NSW Australia; 4 Illawarra Health and Medical Research Institute, Wollongong, NSW Australia

**Keywords:** Food systems, Public health, Local government, Food policy, Public policy, Social determinants of health, Commercial determinants of health, Food swamps, Corporate agnogenesis

## Abstract

**Purpose of review:**

Food systems at all levels are experiencing various states of dysfunction and crisis, and in turn their governance contributes to other intensifying crises, such as climate change, biodiversity loss and the rapid expansion of dietary-related non-communicable diseases. In many jurisdictions governments at local, state and national levels are taking action to tackle some of the key challenges confronting food systems through a range of regulatory, legislative and fiscal measures. This article comprises a narrative review summarising recent relevant literature with a focus on the intersection between corporate power and public health. The review sought to identify some of the principal barriers for the design and support of healthy food systems and environments, as well as key reforms that can be adopted to address these barriers, with a focus on the role of local governments.

**Recent findings:**

The review found that, where permitted to do so by authorising legislative and regulatory frameworks, and where political and executive leadership prioritises healthy and sustainable food systems, local governments have demonstrated the capacity to exercise legislative and regulatory powers, such as planning powers to constrain the expansion of the fast food industry. In doing so, they have been able to advance broader goals of public health and wellbeing, as well as support the strengthening and expansion of healthy and sustainable food systems.

**Summary:**

Whilst local governments in various jurisdictions have demonstrated the capacity to take effective action to advance public health and environmental goals, such interventions take place in the context of a food system dominated by the corporate determinants of health. Accordingly, their wider health-promoting impact will remain limited in the absence of substantive reform at all levels of government.

## Introduction

Whether we examine the factory farming of livestock [or] the proliferation of ultra-processed and unhealthy foods and sugary beverages…the underlying theme is clear: the contemporary global food system has generated a pandemic of non-communicable diseases and produced environmental devastation on a barely comprehensible scale. This sombre picture becomes bleaker still when we examine the multiple intersecting and reinforcing policy, regulatory and institutional mechanisms and dynamics by which this food system further entrenches, consolidates and expands itself, and is being expanded, through time and space…[The COVID-19 pandemic and its handling] has brought into the sharpest possible relief the opposing interests of national and global public health, on the one hand; and the relentless…drive for capital accumulation and profit, on the other—regardless of the consequences. [1]

**Fig. 1:**
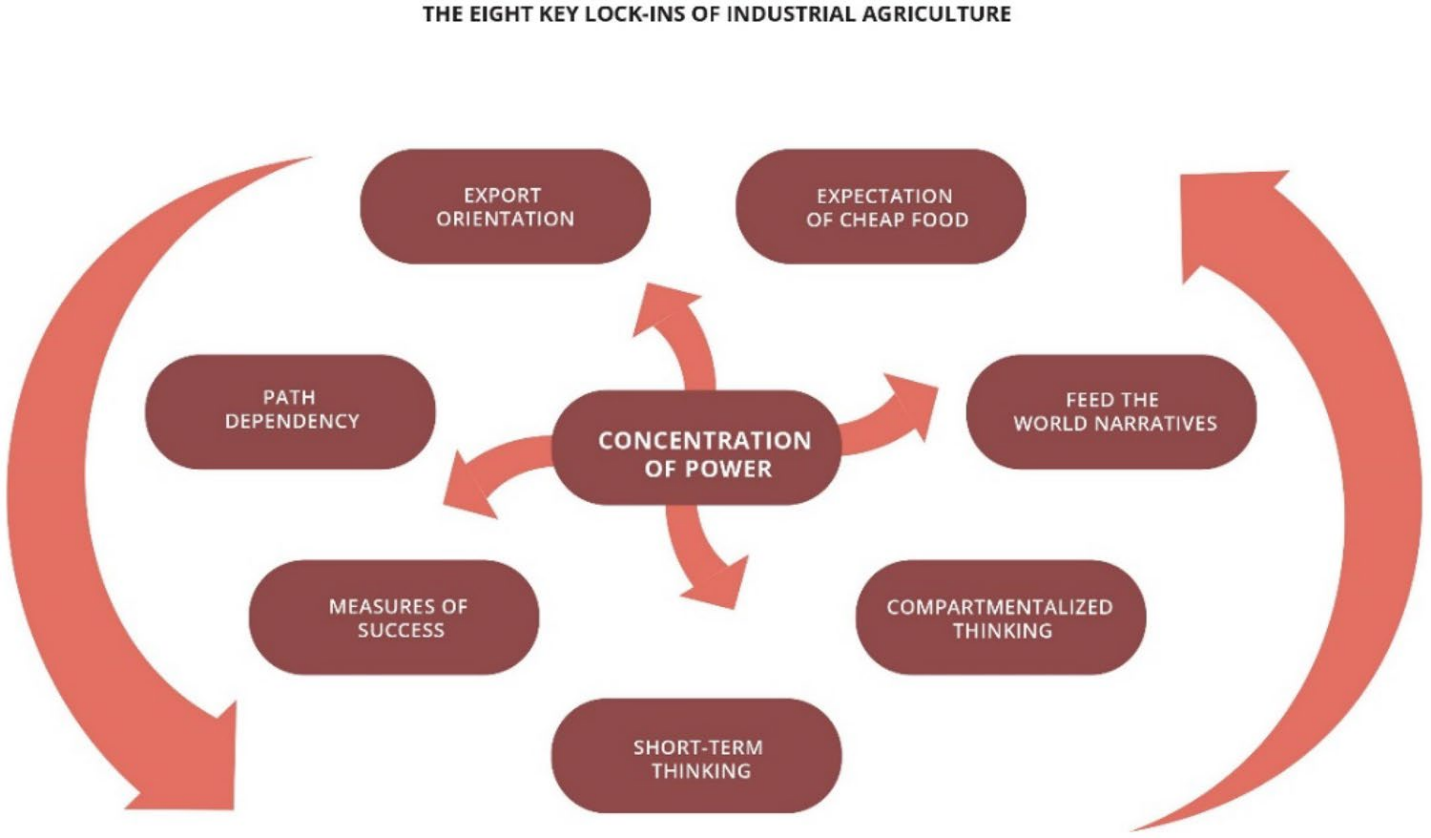
Mooney P. (2017). Too big to feed: exploring the impacts of mega-mergers, consolidation and concentration of power in the agrifood sector. International Panel of Experts on Sustainable Food Systems (IPES-Food). http://www.ipes-food.org/_img/upload/files/Concentration_FullReport.pdf. Reproduced with permission from IPES-Food

The case for transformative change in local, national and global food systems is, we argue, overwhelming. However, enormous obstacles to such transformation exist in the form of: (1) powerful, multinational agrifood corporations who benefit enormously from the status quo (“Big Food”); (2) food industry-focused policy directives that are geared towards manufacture and export of ultra-processed foods; (3) a ‘feed the world’ narrative founded on the expectation of cheap food and techno-optimism, all of which is underpinned by short-term thinking and profit expectations. This is illustrated in Fig. 1, taken from a report by the influential International Panel of Experts on Sustainable Food Systems (IPES-Food) [2] on industry consolidation in the global food system. In this review article, we examine recent scholarship on the ability of local governments to contribute to the change process. We consider policy levers available under urban planning frameworks, whilst also acknowledging the limitations to such efforts by reference to the wider context of food system dynamics and the corporate determinants of health. The article is organised as follows: first, we provide an overview of the emerging framework of the corporate determinants of health. Next, we examine a particularly significant element of this framework, as regards advocacy for legislative and regulatory reform, in the form of ‘corporate agnogenesis’. This is characterised as the deployment of a variety of tactics including the selective and misleading use of evidence to actively introduce doubt and ignorance in policy-making processes regarding the harmful human and ecological health impacts of processed and fast food. In the third section, we examine the ability of local governments to advance healthy and sustainable food system goals citing examples from UK and the USA, contrasted with the city of Melbourne in Australia. Finally, we offer recommendations on actions to pursue a transformative agenda for healthy food systems.

## Corporate Determinants of Health

**Fig. 2:**
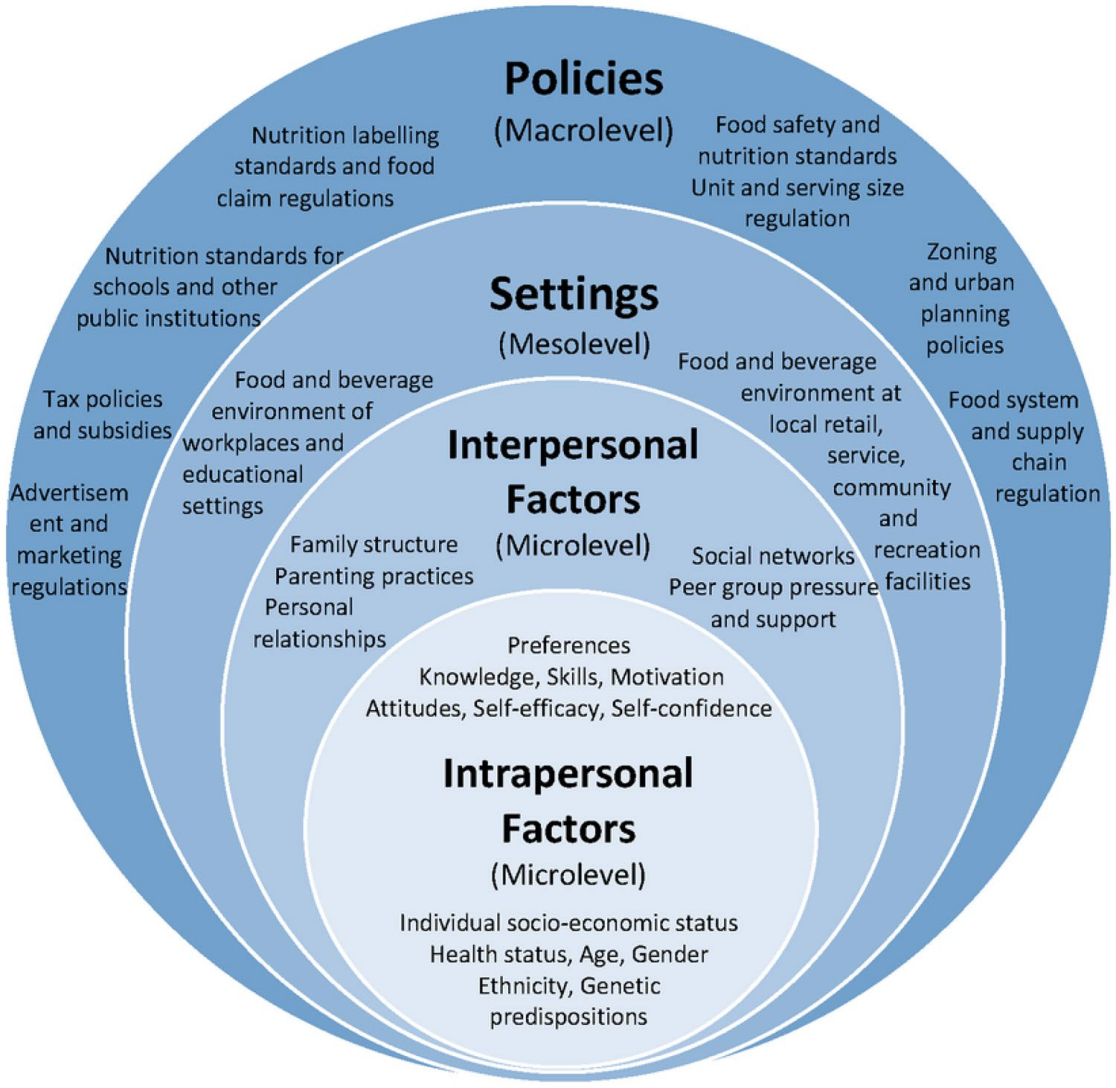
Socioecological model of public health: von Philipsborn P et al., Stratil JM, Burns J, Busert LK, Pfadenhauer LM, Polus S, Holzapfel C, Hauner H, Rehfuess E. Environmental interventions to reduce the consumption of sugar‐sweetened beverages and their effects on health. *Cochrane Database of Systematic Reviews*. 2019(6). Reproduced with permission from the authors

Public health scholars and practitioners are very familiar with the socio-ecological model of public health, with its five levels of the ‘social determinants of health’, beginning with the individual and then progressing through the interpersonal, the organisational, the community and finally to the societal and policy level [3]-see Fig. 2. This framework draws attention to the interdependencies amongst the various levels, the complex interplay amongst a wide array of context-specific factors and the need for a systems-based understanding when designing and implementing policies and programs. At the same time, in the absence of a critical lens, the model can lead to a certain detached and apolitical perspective in which interventions become matters of technocratic design and execution, thus obscuring the effects of corporate interests and actions to shape the social determinants at every level [4•].

**Fig. 3:**
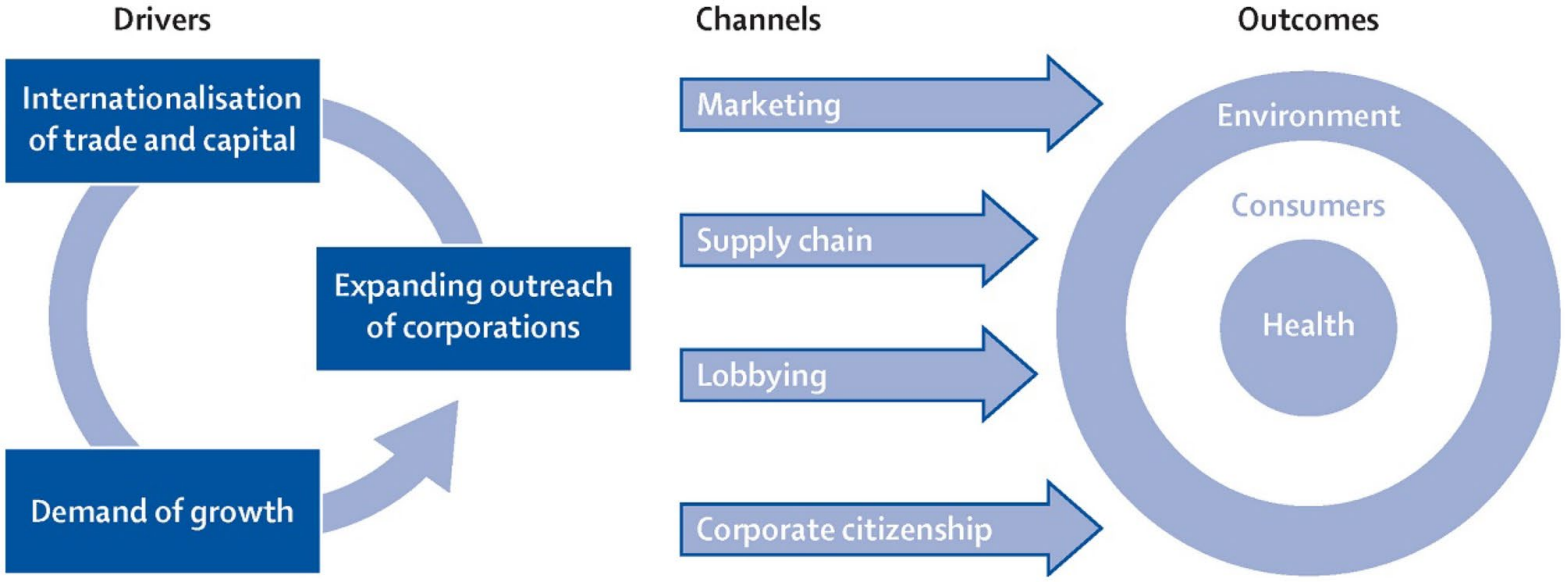
Commercial determinants of health. From Kickbusch I, Allen L, Franz C. The commercial determinants of health. *The Lancet Global Health*. 2016 Dec 1;4(12):e895-6. Republished with permission from the authors

To address such shortcomings, over the past few years a body of research and literature has emerged to consider the social determinants alongside the commercial, or corporate, determinants of health. In one of the first substantive articulations, Kickbush, Allen and Franz [5] (see Fig. 3) defined these commercial determinants as follows:

[S]trategies and approaches used by the private sector to promote products and choices that are detrimental to health. This single concept unites a number of others: at the micro-level, these include consumer and health behaviour, individualisation, and choice; at the macro-level, the global risk society, the global consumer society, and the political economy of globalisation*.* [e895].

When compared with the socio-ecological model of public health (Fig. 2), the commercial determinants of health model has the virtue of focusing attention on the most critical structural impediments to optimal public health. In contrast, the socio-ecological model tends to obscure the locus of power and influence under the general rubric of ‘policy’, and suggests that the actual core of good public health is the intra- and interpersonal factors. However, as Millar [6] has stated simply: ‘[e]ffective prevention of chronic disease requires addressing the corporate determinants of health’, and includes regulatory measures such as limiting the advertising of junk food to children and fiscal measures such as a tax on sugar-sweetened beverages.

A significant body of research now describes the tactics used by Big Food to forestall, weaken, or disrupt effective public health policy. For example, McKee and Stuckler [7•] identified how corporations influence policy through narrative framing, rule-setting, commodification of knowledge, and ‘undermining political, social and economic rights’. Taking the corporate determinants of health framework further, Lencucha and Thow [8] argued that, while important, analysing the strategies deployed by corporate manufacturers and purveyors of unhealthy products to shape the policy environment to suit their commercial interests was by itself insufficient. In order to reveal the reasons for the contradictions and incoherence between government policy objectives that sought to promote good public health, on the one hand, and to facilitate economic growth, expansion of businesses, trade and employment opportunities, on the other, researchers needed to look at the underlying systemic and structural factors that influenced institutional behaviour and policy settings. Echoing the work of IPES-Food (Fig. 1), Lencucha and Thow pointed to the manner in which the ‘dominant neoliberal policy paradigm’ had become institutionally embedded across many national and global policy-making forums as the locus of these systemic drivers. Drawing on the work of critical political economists and human geographers such as David Harvey, Stephen Gill and Gerardo Otero, Lencucha and Thow describe the ‘core tenets’ of the neoliberal paradigm as constituted by a policy agenda of trade liberalisation, privatisation of state-owned utility companies and other public assets, progressive dismantling of welfare state and workers’ rights protections; and the reorientation of government regulatory powers to safeguard corporate interests, valorise the market, and protect private property rights [8]. All of this has been legitimated through a heavily ideological discourse that asserts the primacy of ‘individual rights and freedoms’ and ‘consumer choice’, whilst governments of major economies have created and expanded a policy paradigm in which *inter alia* food industry multinationals have become ‘vectors of non-communicable diseases’ [4•].

In his commentary on their paper, Labonte [9] extended Lencucha and Thow’s analysis by noting how neoliberalism itself had proceeded through various iterations in the past forty years, from structural adjustment and privatisation (1980s) to financialisation (1990s and 2000s), and then to austerity in the wake of the Global Financial Crisis (2010s). He argued that it had now arrived at a new and even more dangerous juncture, characterised by autocratic, nationalistic and even neo-fascistic tendencies. Drawing on Harvey’s [10] insight that, above all, neoliberalism was a class project to restore the power and material status of the owners of capital, and thus one of its key goals was to roll back social gains and redistributive policies of the immediate post-war decades, Labonte suggested that we have arrived, with the fourth iteration of neoliberalism, at a more predatory and rapacious stage of capitalism, and that the task at hand was now to proceed to its ‘thorough transformation’ [9]. In a similar vein, Rose argued that ‘[t]he cause of food system crises is to be found in the core logic of capital accumulation, the profit imperative, and the relentless and expanding processes of commodification and financialisation’ [1].

## Corporate Agnogenesis

Public health policy-makers are of course aware of the commercial determinants of health and many are attempting to take steps to counter them, in particular through stricter regulation of marketing and promotion of unhealthy food and beverage products, and efforts to reformulate these products to be more healthful. Where governments take such measures, they inevitably encounter various strategies of resistance from the corporations that profit from the manufacture and sale of such products.

### Taxes on Unhealthy Foods: Resistance from Industry

The case of sugar-sweetened beverages is illustrative, with a number of countries, such as Mexico, the UK and South Africa, attempting to reduce the consumption of these products through the imposition of a ‘sugar tax’. Everywhere such a tax has been introduced, or its introduction attempted, it has been the subject of vigorous corporate lobbying in opposition—consistent with what would be expected from the commercial determinants of health framework. One such corporate tactic is what Gary Fooks and his colleagues, in their examination of submissions made to the South African government regarding a proposed sugar tax, term ‘agnogenic practices’ [11••]. Such practices ‘refer to methods of representing, communicating and producing scientific research and evidence which work to create ignorance or doubt irrespective of the strength of the underlying evidence’ [11••, 12•].

**Fig. 4:**
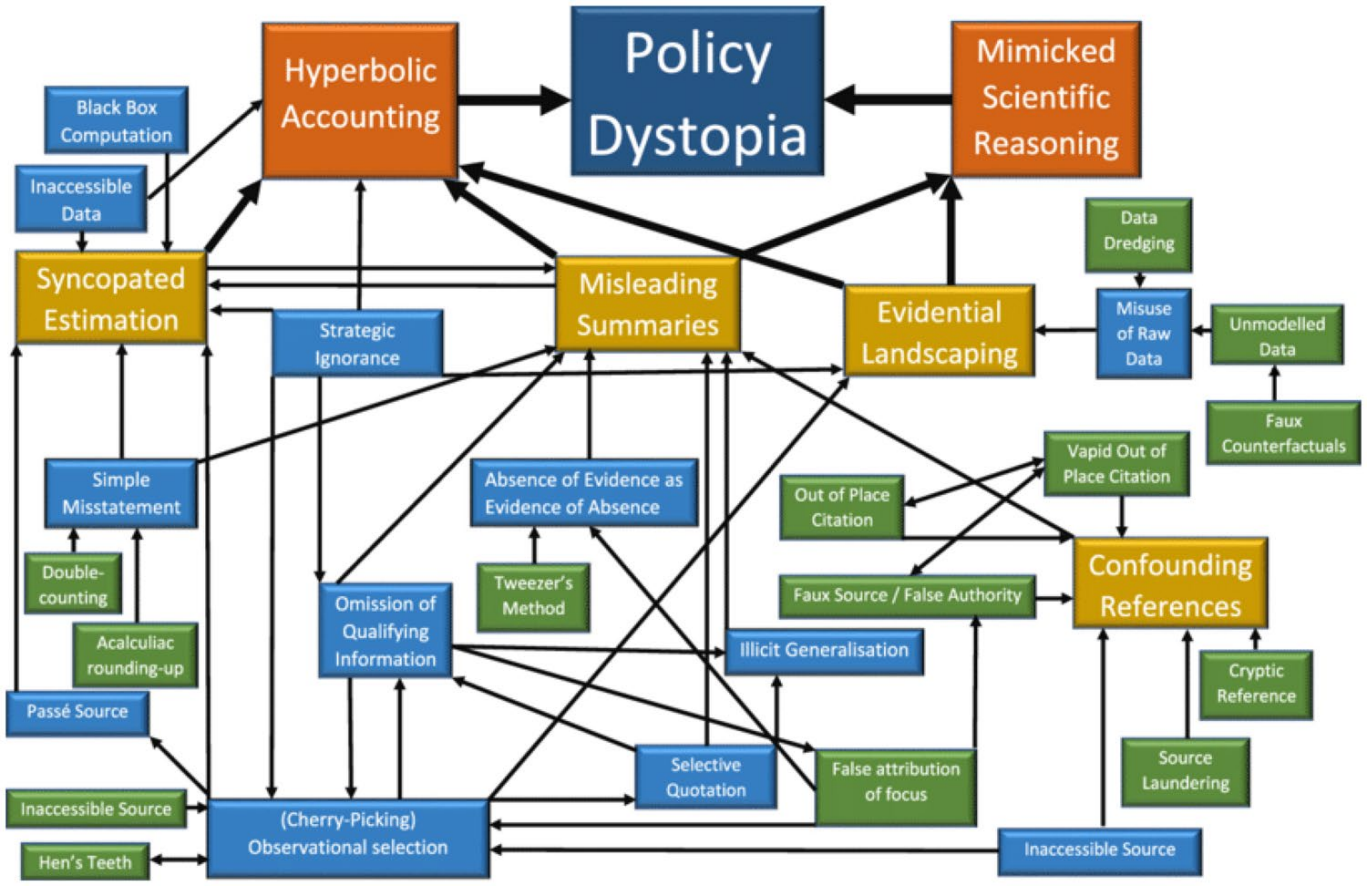
Model of Corporate Agnogenesis of Soft Drink Companies in the context of South Africa's Consultation on a Proposed Taxation on Sugar-Sweetened Beverages: Fooks, G.J., Williams, S., Box, G. et al. Corporations’ use and misuse of evidence to influence health policy: a case study of sugar-sweetened beverage taxation. *Global Health* 15, 56 (2019). Reproduced with permission of the authors

Fooks et al. detected four distinct strategies in their analysis of industry submissions, which they grouped as: a) ‘confounding referencing and misleading summaries’; b)‘misuse of raw data’; c) ‘evidential landscaping’ including cherry-picking and selective quotations; and d) ‘hyperbolic accounting’ to build an overarching narrative of ‘policy dystopia’ that exaggerated the impact of a sugar tax on the loss of jobs and harms to economic development [11••] (see Fig. 4). Within each of these broad categories, the authors detected no fewer than 23 distinct sub-categories of agnogenic practices, such as ‘source laundering’, ‘false attribution of focus’, ‘illicit generalisation’, ‘double-counting’, the ‘hen’s teeth method of cherry-picking’ and ‘black box computation’ [11••]. The authors note how ‘scientific uncertainty’ which, by the very nature of scientific research and the progressive advancement of human knowledge, is almost inevitable, ‘highlight[s] the structural vulnerability of modern modes of evidence-based policy-making to corporate agnogenesis’ [11••].

Earlier work by Emilia Sanabria built on literature examining the ‘social production of ignorance’ to show how the food industry has been ‘highly active’ in ‘[deliberately forging a consensus] concerning the uncertainty and complexity of obesity’s determinants [to obscure its own role and deflect] responsibilities back to the purported knowledge deficiencies of individual eaters’ [12•]. In so doing, she also brought directly into critical consideration the meaning of ‘evidence’ and ‘evidence-based policy’, arguing that because of the ‘heavy over-determination’ of the ‘myriad nested interactions’ that collectively constitute the phenomenon of obesity, the ‘epistemic regimes that dominate public health…frame complex problems in a manner that reduces them to what is manageable, even when such framings are…shown to be inadequate’ [12•]. In public health policy terms, the effect has been a marked ‘disproportion between exhortations to healthy eating aimed at individual consumers and [a] remarkable laissez-faire that characterises how the food and drink industries are invited to voluntarily limit fat, sugar or salt in their products’ [12•]. Again, this is consistent with an epistemic framing of public health within a largely de-politicised socio-ecological model in which corporate power and influence is rendered invisible and the primary focus of interventions are aimed at the intra- and interpersonal levels (Fig. 2).

## Ability of Local Governments to Advance Healthy and Sustainable Food Systems and Environments

Undoubtedly, upstream policies made at national or federal level, as well as state or provincial levels, are highly influential in shaping the healthfulness and sustainability of the food system. However, local governments also have a potentially important role in advancing the transformation of existing food systems, including through planning law and policy.

### Urban Planning and the Commercial Determinants of Health

Urban planning is primarily a state government responsibility in Australia, with legislation in each state and territory creating the planning frameworks that regulate land-use development. However, under this legislation, local governments are delegated a significant role in day-to-day planning decision-making, including approving most development applications. Such decision-making must, however, be made in accordance with the principles and directives emanating from State planning legislation and policy (which may differ according to individual state). From the perspective of promoting public health in general, and healthy and sustainable food systems in particular, there is a large legislative obstacle in the fact that planning frameworks explicitly prioritise competition principles and market freedoms when decisions are to be made regarding the establishment of new businesses selling harmful or potentially harmful products. Conversely, state planning legislation has not included health promotion as an objective, effectively preventing local governments from taking public health into account in planning decision making [13]. It may be for this reason that the public health impact of fast food restaurants is rarely mentioned in planning decisions made by Australian local governments. There is limited academic research on the role of Australian local government planning decisions in regulating unhealthy food retail. However, Muhunthan and colleagues’ [14•] analysis of 44 cases of judicial review (2010–2015) of administrative decisions made by Australian local and state governments regarding the opening of new alcohol outlets illustrates how the planning scheme prioritises industry interests over those of public health. The authors found that more than three-quarters of the judicial decisions (77%) were favourable to the interests of industry [14•]. Importantly, ‘public health research evidence appeared to have little or no influence’ on the outcomes of cases, given the legislative voids noted above. The authors noted how the so-called ‘hot-tubbing’ of expert evidence (i.e. where expert witnesses from both sides submit a ‘joint report outlining matters on which they agreed and disagreed’) almost invariably worked to the benefit of industry applicants, who were able to make use of data they claimed was ‘locally relevant’ [14•]. In contrast, local governments defending decisions to refuse permission for new liquor outlets on the basis of their Municipal Public Health and Wellbeing Plans (a local government health planning instrument mandated by state government legislation in Victoria) and / or by reliance on academic evidence of the harmful effects of alcohol consumption in general rather than in relation to the specific locality, appeared to be given short shrift in the judicial decisions [14•].

Such tactics by alcohol industry litigants would appear to be another example of corporate agnogenesis in form of the ‘confounding references’ and ‘evidential landscaping’ [11••], contributing to a marked tendency by judicial officers to disregard or minimise extensive public health evidence. This evidence generally related to the harmful effects of *inter alia* underage drinking in Australia, with industry experts being adept at casting doubt as to whether any locally relevant causal links between such tendencies and the liquor outlet in question existed [14•]. The same judicial decision-making process is also observable in the few cases that have been litigated regarding the opening of new fast food restaurants in Australia. The most famous is the *McDonalds v Yarra Ranges Shire Council* 2012 decision of the Victorian Civil and Administrative Tribunal [15]. In that case, the fast food company sought to open a new, 24 h-a-day dine-in and drive-through restaurant directly opposite a primary school and pre-school centre. Over 1300 objections were lodged by local residents and the schools in question on various grounds, including the anticipated health impacts of the restaurant on the local population and its school children, and a well organised residents’ campaign opposing the opening of the restaurant was mounted over many months. However, VCAT summarily dismissed these objections, including that of public health, as being irrelevant, on the narrow grounds that the site in question was classified as a Business Zone 1 within the local Planning Scheme and therefore no planning permission was required for the operation of a convenience restaurant as such (i.e. a convenience restaurant was an ‘as of right’ use in a Business Zone). The determination regarding the planning permission was considered by VCAT to be distinct from ancillary matters such as parking, lighting, signage and landscaping in the construction of the restaurant [15].

In her review of 33 cases involving fast food-related planning appeals in Victoria over the period from 1969–2012, Taylor [16•] noted continuing community opposition to McDonalds and other fast food franchises such as Kentucky Fried Chicken (KFC), as well as key moments in the standardisation of planning provisions at the state level, especially in the early 1990s, that have made the expansion of this industry almost inevitable [16•]. Significantly, two key amendments were made in 1992 and 1994 to replace existing terms such as ‘takeaway food’, ‘drive-throughs’ and ‘service premises’ with the catch-all phrase ‘convenience restaurants’. These changes were made specifically at the request of McDonalds Australia Limited and the panel report advising the Victorian State Government on the recommended amendments expressly stated that the motive behind them was ‘to remove disincentives to the future expansion of the fast food industry within metropolitan Melbourne’ [16•]. The amendments permitted franchised restaurants to redefine themselves into a broader category of uses in order to avoid negative public opinion from harming their ability to expand at a rapid rate. This is a clear illustration of the concept of the corporate determinants of health: the State Government modified existing planning laws in a way that favoured industry interests, at the expense of public health [7•], particularly given the demonstrated association between the number of fast-food restaurants in a particular area and poor diet-related health outcomes (as described below). Further, as a result of these changes under the Victorian Planning Provisions, democracy in the decision-making process at the local council level has been removed, since the number of objections by residents and other interested parties now has no legal relevance to overall outcomes for planning permission applications of ‘convenience restaurants’ [16•].

In light of the continuing expansion of the fast food industry as well as the worsening obesity epidemic, urban planning scholars are calling for urgent reforms to Australia’s State planning provisions to strengthen local governments’ ability to consider public health concerns in applications for new fast food outlets. Suggested reforms include amendments that will explicitly make public health a relevant consideration (along with environmental and amenity concerns), and place the burden of proof to demonstrate ‘no harm’ to residents’ health on the applicant businesses, rather than it being the responsibility of the objecting residents and other stakeholders to substantiate their objections, as is currently the case [17]. However, these planning challenges cannot be addressed by Australian local governments alone, as they are compounded by the lack of effective state and federal government controls over the fast food industry in relation to other corporate determinants of health. These include product marketing and sports sponsorship, as well as a reform of urban development models for new suburbs that are premised on the assumption that town centres will consist of supermarkets, ‘big box’ stores, fast food restaurants and ancillary retail outlets all located in shopping centres that are primarily, if not solely, accessible by car.

### Food Systems and the Built Environment: The Emergence of Food Swamps

The rapid expansion of the fast industry is now described as the ‘food swamp’ phenomenon, understood as a ‘spatial metaphor to describe neighbourhoods where there is a higher density of food outlets selling unhealthy quick serve foods, which are energy dense and nutrient poor, relative to the density of food outlets selling healthy options’ [18••]. Research indicates that food swamps tend to impact lower-income areas, and in particular growth-area suburbs on the outer fringes of sprawling cities such as Melbourne and Perth; and that they tend to be moderate to strong predictors of obesity rates [17, 19]. Analysis for Melbourne’s growth-area suburbs shows that food swamp prevalence has increased by 92% over the decade 2008–2018, with an average of nine unhealthy food outlets for every one healthy food outlet [18••]. An earlier analysis of the location in Melbourne of 537 fast food restaurants from four major chains found that there was ‘greater locational access to fast food restaurants in more socioeconomically disadvantaged areas [and] nearby to primary and secondary schools within the most disadvantaged areas of the major city region’ [20]. Similar observations have been made in New Zealand [21], Winnipeg, Canada [22] and Perth, Western Australia [23].

The issue is becoming more acute as cities struggle to provide affordable housing, with new suburbs located on the urban fringe, dozens of kilometres from the city centre where most employment is concentrated. In the case of Melbourne, Australia’s second-largest and most rapidly growing city, more than half of the anticipated 60% increase in the city’s population over the next 20 years is expected to be concentrated in its belt of ‘growth area’ municipalities on the outer urban fringe. A principal beneficiary of the new retail food environments created by such expansion is the fast food industry: McDonalds Australia achieved revenue of $1.68 bn in 2019, out of a total industry size of $20.6 bn; with industry reports forecasting a compounding annual growth rate for the sector as a whole of nearly 5% in the five years to 2026, having regard to Australians’ preferences for eating out at least twice a week as well as the growth of the home delivery takeaway market [24].

Public policy and planning recommendations to address food swamps range from the creation of incentives for the early establishment of supermarkets and healthy food outlets as new suburbs are established in growth areas [23] to more robust planning measures restricting the density of fast food outlets [21]. A recent systematic review examining the relationship between unhealthy food environments and rates of obesity in the USA and several other countries found a clear association in all countries between lower socio-economic neighbourhoods, high concentrations of unhealthy food outlets, higher consumption of unhealthy food and higher rates of obesity [20, 25]. Conversely, there is a positive association in Australian capital cities between supermarket availability and healthful body size [26].

### Local Government Regulation of Fast Food Outlets in England and the USA

While local governments in Australia do not presently have the power to control the expansion of the fast food industry on public health grounds, this is not the case in other jurisdictions. In particular, local authorities in England have the ability, under national planning policy, to impose stricter regulation of fast food outlets. The 2012 National Planning Policy Framework and associated guidance requires local planning authorities to ‘promote healthy communities’, ‘work with public health leads and organisations to understand and take account of the health status and needs of the local population’, and to ‘promote access to healthier foods’ [27]. A census of 325 local government areas in England conducted in 2018 revealed that just over half (164) ‘had a policy specifically targeting takeaway food outlets’, with just over a third of those (56) focusing on health [28••]. The creation of ‘exclusion zones around places for children and families’ (e.g. schools) was the ‘most common health-focused approach’ [28••]. The same group of authors subsequently conducted a further qualitative study of planning and public health professionals in English local authorities in order to explore their experiences with such regulatory measures and the criteria for their successful adoption and implementation [29••]. Key findings identified that: local government planning policies were primarily driven by health outcomes with a strong focus on prevention of childhood and youth obesity; regulation of take-away food outlets were ‘seen as *low hanging fruit* because of their segregation from other food outlets in the Use Class Order’ (4); planning professionals ‘felt empowered to regulate’ these outlets because of the tools they had been given which created a ‘perceived obligation to act’; guidance, precedents and local evidence were important in justifying regulatory measures; and ‘cross-department collaboration [between planning and public health professionals] facilitated adoption’ (5). Challenges included objections from the fast food industry and ‘nanny-state’ criticisms from local politicians. The adoption of the new controls has resulted in a reduction in the opening of new take-away food outlets.

Earlier, in 2008, a ‘fast food ban’ had been enacted by the City of Los Angeles, prohibiting the construction of new ‘stand-alone’ fast food outlets in low-income neighbourhoods in South Los Angeles [30••]. An analysis several years after the ban was introduced showed that, contrary to the expectations of public health proponents of the ban, rates of overweight and obesity in South Los Angeles had continued to increase as had rates of consumption of fast food [30••]. The reasons for this included the limited nature of the ban (prohibiting new stand-alone restaurants rather than restaurants in high street shopping centres) as well as the lack of any broader attempts to shift the prevailing food culture and pricing structure [30••].

The Los Angeles fast food ban is one example of a broader shift embraced by many cities in the USA towards what is termed ‘New Urbanism’ and ‘Smart Growth’ strategies that seek to ‘reduce reliance on automobiles and promote community integration through higher density development, public transportation, public green spaces, pedestrian-friendly development and mixed-use neighbourhoods’ [31]. In the Australian context, an illustration would be the stated goal of’20-min neighbourhoods’ in *Plan Melbourne* (2017), under which residents should be able to satisfy the majority of their daily living and working requirements within a 20-min walk, bike ride or public transport trip from their home [32]. Regarding access to healthy food, public health-focused zoning and rating policies have been introduced in various US cities to attract grocery stores and supermarkets into areas classified as ‘food deserts’, as well as to encourage and incentivise various forms of urban agriculture [31]. These measures illustrate how restrictions on fast-food restaurants need to be accompanied by planning, regulatory and fiscal strategies to improve a neighbourhood’s walkability and the availability of public transport, as well as encouraging urban agriculture and healthy food retail, to create a truly healthy food environment. However, in response to these and related non-land use measures (such as the prohibition of ‘happy meals’ by San Francisco), the fast food industry has used its lobbying power at the State government level in various instances to limit the capacity of local governments to act [31], again demonstrating the challenge of managing corporate influences on public health policy, including at the local level.

## A Necessary Reform Agenda to Tackle the Commercial Determinants of Health for Food Systems Transformation

It is unsurprising that corporations have strenuously resisted the stricter regulation of highly profitable processed foods such as sugar-sweetened beverages (SSBs), given that evidence is emerging that regulation, where it occurs, is proving effective in terms of reducing consumption of these products [33]. In their survey of the seven US cities and 40 countries where such measures have been introduced, Krieger and colleagues found that the most effective interventions are sugar taxes and front-of-pack health warnings, followed by restrictions on marketing to children and food service guidelines in public educational and health institutions such as schools, hospitals, prisons and universities [33]. The authors classify the policy interventions into four categories: financial (taxes, restrictions on discounts); information / advertising (front of package warnings, advertising warnings, marketing restrictions); defaults (mandatory healthy beverages for children); and availability (healthy retail, procurement) [33]. In relation to sugar taxes, a review of the evidence across several US jurisdictions as well as the UK and South Africa indicated that the implementation of such taxes has led to reductions in the consumption of SSBs ranging from 21 to 38%, increases in revenues for local governments that have been invested in health measures benefiting low-income communities, no disproportionate impact on lower income groups, and no discernible loss of jobs [33]. The authors cite Chile’s Food Labeling and Marketing Law (2012) as a standout example of the effectiveness of mandatory front-of-pack health warnings, with ‘[p]urchases of beverages with “high-in” labels [falling] by 23.7% after implementation, with similar reductions across all income groups’ [33]. More broadly, this legislation is highlighted as ‘an excellent example of policy integration’, with ‘[restrictions] on child-directed marketing [and bans of] sales in schools of foods and beverages high in added sugars, sodium or saturated fats’, with implementation accompanied by guidance to all stakeholders ‘as well as mass media campaigns on using the warning labels’ [33].

Experience with regulatory and policy measures to reduce tobacco consumption is instructive and could be applied to reform in food systems governance. By the end of the twentieth century, smoking had caused 100 million deaths worldwide and, in the absence of effective control efforts, was predicted to cause 1 billion entirely preventable deaths by the end of the twenty-first century [34•]. An internationally-coordinated effort was marshalled under the World Health Organisation’s (WHO) Framework Convention for Tobacco Control (FCTC) (2005), and has been adopted by ‘181 WHO member states and the European Union, thereby covering more than 90% of the world’s population’ [34•]. The implementation of the Convention took place under the ‘MPOWER package’, consisting of ‘the six best-practice cost-effective interventions defined in the FCTC, namely ‘Monitor tobacco usage and prevention policies’ (M); ‘Protect people from tobacco smoke’ (P); ‘Offer help to quit tobacco use’ (O); ‘Warn about the dangers of tobacco’ (W); ‘Enforce bans on tobacco advertising, promotion and sponsorship’ (E); and ‘Raise taxes on tobacco’ (R) [34•]. By 2021, ‘about 5 billion people living in 136 countries, equivalent to 65% of the world’s population, are…benefiting from at least one of these MPOWER measures implemented at the highest level…a fivefold increase from the 1.1 billion people benefiting from tobacco control measures back in 2007’ [34•]. The most effective measures, yet the least implemented, are cigarette taxes; and ‘only four countries (Australia, Turkey, the UK and Vietnam) have run best-practice mass media campaigns repeatedly since 2010’ [34•]. The evidence suggests that these policies work, with a nearly 6% reduction in ‘prevalence of current tobacco smoking…since the beginning of the twenty-first century’, and an increase to 21.1% of the proportion of people protected by smoke-free policies in 2018, up from just 3% in 2007 [34•].

In terms of further accelerating the decline in smoking, Peruga et al. highlight three measures in particular: a) ‘significantly increase real prices of all tobacco products through tobacco taxes’; b) ban industry tactics to ‘engineer the attractiveness of tobacco products [such as] characterising flavours’; and c) ‘ban the most insidious form of tobacco promotion: Corporate Social Responsibility’ [34•]. Implementing such measures in the face of continued, sophisticated and determined industry opposition will not be easy, however doing so requires ‘overcoming the false health versus economy dilemma’ and fully acknowledging that ‘the interests of the tobacco industry are irreconcilable with tobacco control and public health’ [34•].

## Conclusion

With diet now overtaking tobacco as the leading cause of ill-health and disease in many countries, including Australia, the lessons of the experience with tobacco harm reduction policies, as well as the experience to date of policy and regulatory efforts to reduce the consumption of SSBs and junk food, are clear. The MPOWER framework is a valuable precedent that could be adapted and applied to reduce harm from unhealthy foods and beverages. Facing down industry arguments about the alleged economic impacts of effective public health measures is essential. Taxation and price increases of unhealthy and addictive products work; and the revenue raised can be, and is being, invested in low-income communities to improve health outcomes and enhance quality of life. Prominent front-of-pack warnings and mass media campaigns are also effective, as is the banning of sponsorship of major sports and denying harmful industries the ability to burnish their public image through so-called corporate social responsibility. In the growth area context of sprawling cities in Australia and elsewhere, reforms to the planning framework to empower local governments to refuse applications for new fast food restaurants and thereby reduce the food swamp phenomenon that plagues these suburbs is clearly in accordance with good public health policy. It should, we argue, form part of an equivalent and adapted MPOWERR framework for transforming the contemporary food system into one that more fully promotes health, equity and environmental sustainability (see Table 1). Ultimately, however, political will is required to overcome the corporate determinants of health and the effect of corporate agnogenesis on food systems-including at the local level**-**in order to govern in a manner consistent with optimum public health outcomes.

**Table 1:** Summary of reform measures for SSBs, tobacco and unhealthy food

**SSB Policy Interventions**—Krieger et al	**MPOWER**—WHO Framework Convention on Tobacco	**MPOWERR**—Food system reform recommendations
**Financial** e.g. taxes, restrictions on discounting	**Monitor** usage	**Monitor** consumption of unhealthy foods and beverages (including fast food) and rates of obesity / ill-health
**Information / Advertising** e.g. warning labels	**Protect** from tobacco smoke	**Protect** children and youth from marketing for unhealthy foods and beverages
**Default provision**—mandatory healthy options	**Offer** support to quit	**Offer** healthy food retail, food growing spaces, etc
**Availability**—procurement, healthy retail	**Warn** about dangers	**Warn** about dangers of unhealthy foods/beverages with mandatory front-of-pack labelling
	**Enforce** bans on advertising / sponsorship	**Enforce** bans on advertising / sponsorship by fast food restaurant companies and other food companies with portfolios of mainly unhealthy products
	**Raise** taxes on tobacco	**Raise** taxes on unhealthy & addictive food and beverage products / restrict discounting
		**Reform** State planning provisions to restrict the opening of unhealthy food retail outlets and encourage the opening of healthy food retail outlets
